# Graphene Oxide promotes embryonic stem cell differentiation to haematopoietic lineage

**DOI:** 10.1038/srep25917

**Published:** 2016-05-20

**Authors:** Eva Garcia-Alegria, Maria Iluit, Monika Stefanska, Claudio Silva, Sebastian Heeg, Susan J. Kimber, Valerie Kouskoff, Georges Lacaud, Aravind Vijayaraghavan, Kiran Batta

**Affiliations:** 1 Cancer Research UK Stem Cell Hematopoiesis Group, Cancer Research UK Manchester Institute, University of Manchester, Manchester, United Kingdom; 2 School of Materials and National Graphene Institute, University of Manchester, UK; 3 Cancer Research UK Stem Cell Biology Group, Cancer Research UK Manchester Institute, University of Manchester, Manchester, United Kingdom; 4 Faculty of Life Sciences, Michael Smith Building, Manchester, United Kingdom

## Abstract

Pluripotent stem cells represent a promising source of differentiated tissue-specific stem and multipotent progenitor cells for regenerative medicine and drug testing. The realisation of this potential relies on the establishment of robust and reproducible protocols of differentiation. Several reports have highlighted the importance of biomaterials in assisting directed differentiation. Graphene oxide (GO) is a novel material that has attracted increasing interest in the field of biomedicine. In this study, we demonstrate that GO coated substrates significantly enhance the differentiation of mouse embryonic stem (ES) cells to both primitive and definitive haematopoietic cells. GO does not affect cell proliferation or survival of differentiated cells but rather enhances the transition of haemangioblasts to haemogenic endothelial cells, a key step during haematopoietic specification. Importantly, GO also improves, in addition to murine, human ES cell differentiation to blood cells. Taken together, our study reveals a positive role for GO in haematopoietic differentiation and suggests that further functionalization of GO could represent a valid strategy for the generation of large numbers of functional blood cells. Producing these cells would accelerate haematopoietic drug toxicity testing and treatment of patients with blood disorders or malignancies.

Bone marrow transplantations are well-established cellular therapies for the treatment of a variety of malignant or genetic disorders of blood cells^1^. The success of these transplantations relies on a rare population of haematopoietic stem cells (HSCs), which can reconstitute the entire blood and immune system cells. However, a major restriction to the wider application of these curative treatments is the difficulty, or even the possibility, to find a healthy source of donor tissue that is immunologically compatible. In the absence of well-matched donors, the use of allogeneic transplantations is often associated with increased morbidity and mortality accompanying graft rejections^2^,^3^. The scarcity in matched donors could potentially be overcome in the future by the provision of unlimited and renewable sources of HSCs from pluripotent stem cells such as embryonic stem cells (ESCs) or patient derived induced pluripotent stem cells (iPSCs)^4^,^5^. Similarly, differentiation of pluripotent stem cells (PSCs) could represent a sustainable source of red blood cells and platelets for transfusions^6^,^7^. The fulfilment of these promises relies on a better understanding of the molecular and cellular mechanisms underlying the development of the haematopoietic system and the establishment of improved protocols of differentiation of pluripotent stem cells toward the blood lineage.

The *in vitro* ESC-derived haematopoietic lineage specification is initiated with a mesodermal-derived precursor termed the blast colony forming cell (BL-CFC), which is the *in vitro* equivalent of the haemangioblast, a mesodermal progenitor with both endothelial and haematopoietic potential^8^,^9^. These BL-CFCs express the mesodermal marker BRACHYURY and foetal liver tyrosine kinase FLK^10^,^11^. Haematopoietic progenitor cells are generated from haemangioblasts through an intermediate haemogenic endothelial population, a specialized endothelium giving rise to haematopoietic cells^12^,^13^,^14^. Similarly to this first wave of haematopoietic development that corresponds to transient yolk sac haematopoiesis, haematopoietic cells that will sustain the adult blood system are also derived from haemogenic endothelial cells present within the lining of dorsal aorta of the embryo^15^,^16^. Although a broad range of haematopoietic cell types such as erythrocytes, myeloid cells and lymphoid cells are routinely derived from ESCs or iPSCs, our current protocols are unable to support the generation of the large numbers of mature functional cells required for clinical purposes^17^. Therefore, there is a compelling need for improved methods of ESCs differentiation to functional blood cells.

Small molecule modulators of signalling pathways and epigenetic modifiers can exert profound effect on the maintenance and differentiation of ESCs^3^. The action of key cytokines, growth factors and modulators along with physical and mechanical stimuli also regulates normal developmental pathways. Activating these signalling pathways in a timely fashion recapitulating normal *in vivo* development would be essential to generate fully differentiated and functional cells^18^,^19^. Indeed sequential modulation of multiple signalling pathways during the course of human ESC differentiation has been recently reported to result in dramatic improvement in the generation of much more functional pancreatic β cells^18^. In addition, growing evidences indicate that biomaterials with their unique ability to mimic *in vivo* architecture and microenvironment provide novel opportunities for the directed differentiation of PSCs to desired lineage^20^,^21^. Three-dimensional (3D) scaffolds in combination with suitable culture conditions should offer efficient methods for the differentiation of ESCs to a desired lineage^22^. New evidences have highlighted the impact of different biomaterials on the maintenance, expansion and differentiation of haematopoietic stem and progenitor cells^23^,^24^,^25^. Similarly, porous biomimetic 3D significantly promote haematopoietic differentiation ability of ESCs^26^. However, studies of the role of biomaterials in generating early haematopoietic progenitors from ESCs have remained limited^27^.

Graphene and related materials have been shown to be excellent supporting scaffolds for PSC differentiation due to their mechanical stability and physiochemical properties^28^,^29^. Graphene oxide (GO), which is derived from the two-dimensional carbon lattice of graphene, has numerous promising applications in biological systems such as drug delivery, gene delivery, and biosensors^30^,^31^. Multi-layered GO can be used for sequential release of molecules of interest and therefore has potential applications in drug delivery^32^. Conjugate of polyethylene amine with GO could effectively transfect plasmid DNA into mammalian cells^31^. GO has also been shown to support ESC growth, to allow spontaneous differentiation and to promote selective differentiation of progenitors. While graphene inhibits the differentiation of ESCs to endoderm, GO in contrast promotes their differentiation to endoderm^33^. In addition graphene and GO promote the differentiation of ESCs to cardiomyocytes and neurons and the differentiation of mesenchymal stem cells (MSCs) to osteogenic lineages^34^,^35^,^36^. Furthermore GO suspension induces neuronal differentiation of neuroblastoma Sh-Sy5Y cells^35^, whereas GO-doped poly(lactic-co-glycolic acid) (PLGA) nanofiber scaffolds promote proliferation and osteogenic differentiation of human mesenchymal stem cells (MSCs)^36^. Finally recent reports have shown that carboxylated GO immobilized with antibodies could be used to capture circulating tumour cells in peripheral blood^37^ and selectively eliminate cancer stem cell population by promoting their differentiation^38^. Mechanistically, GO interacts with cell surface receptors and/or is taken up by cells thereby modulating biological processes^39^. Collectively these results highlight the potential relevance of GO-based biomaterials for future biomedical applications.

In this study, we investigated the relevance of GO in ESC-derived haematopoietic development and established that GO significantly enhances the generation of haematopoietic progenitor cells from both murine and human ESCs. More specifically we determined that GO enhances the transition of haemangioblast to haemogenic endothelium and thereby induces both primitive as well as definitive haematopoiesis.

## Results

### GO coated cover-slip substrates

The GO used in this study was comprised of 100% monolayer flakes (~1 nm thick) and 5–30 μm in lateral size, as determined by atomic force microscopy (AFM) and scanning electron microscopy (SEM). The excellent distribution of 100% monolayer GO flakes which we have obtained in our oxidation and exfoliation procedure is evidenced by the uniform 1 layer step-height in AFM as well as the uniform secondary electron contrast of the flakes seen in SEM. In order to obtain a statistically significant sampling, we combined random SEM and AFM imaging and analysis to cover a large number of flakes with SEM secondary electron contrast, and to validate the SEM contrast using AFM analysis, which is a more direct measure of thickness but can only cover smaller regions in each scan. The C/O ratio in the GO was ~1 as determined by X-ray photoelectron spectroscopy (XPS) and Raman spectroscopy D/G peak intensity ratio was ~0.9. GO coated glass coverslips were used for this study; the GO coating had 100% surface coverage and thickness varied from 10 to 15 nm, indicating few-layered uniform GO deposition (Figs S1–S4, supporting online information).

### GO promotes differentiation of mesoderm to blood progenitors

**Figure 1:**
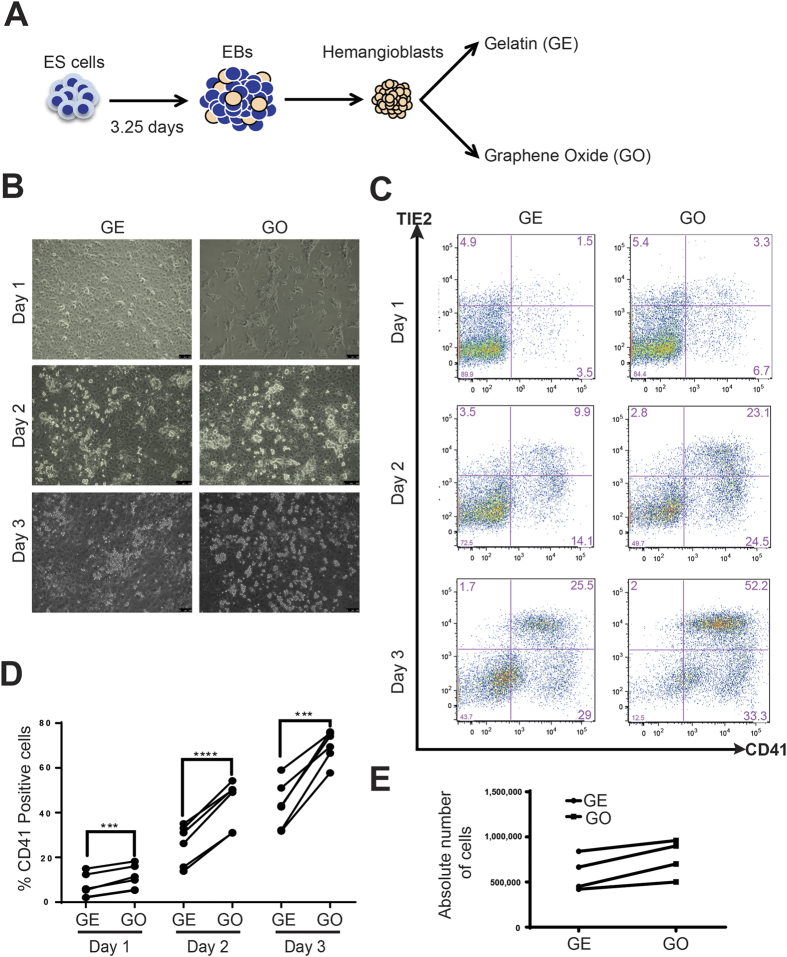
Role of GO on haematopoietic specification. (**A**) Schematic representation of experimental strategy. Haemangioblasts were cell sorted from 3.25-day-old ES derived embryoid bodies and were seeded on to either gelatin (GE) coated plastic plates or graphene oxide (GO) coated coverslips. At days 1, 2 and 3 cells were harvested and analysed for cell surface markers by FACS or clonogenic potential by CFU assays. (**B**) Brightfield images of haemangioblast cultures grown on either GE or GO at indicated days. (**C**) FACS analyses of haemangioblast cultures grown on either GE or GO at indicated days. (**D**) Percentages of CD41 positive cells in haemangioblast cultures grown on either GE or GO at indicated days (Mean, N = 6). (**E**) Absolute number of cells counted in haemangioblast cultures grown on either GE or GO at day 3 (N = 4). Scale bars represent 100 μm. Asterisks indicate significant differences (Paired t-test. ***p < 0.001, ****p < 0.0001).

The role of GO and graphene on the early differentiation of ESCs to different germ layers has been previously reported^33^. In this study, we aimed to examine the role of GO at a later stage during haematopoietic progenitors generation from haemangioblasts, the first mesoderm precursors committed toward the blood program. For this, we differentiated murine ESCs as embryoid bodies for 3.25 days and dissociated them into single cell suspensions. The cells were then enriched for the mesodermal haemangioblast precursor by flow cytometry sorting based on the expression of the tyrosine kinase receptor FLK1 (VEGF–R2). The enriched haemangioblast cell population was then cultured on GO coated cover slips in blast cell culture media or on standard gelatin (GE) coated plates as a control (Fig. 1A). Morphological observation of the cells at day 1 revealed an increase in the formation of cell clusters in cultures on GO in comparison to control GE (Fig. 1B). At later time points, increased emergence of round haematopoietic cells in GO coated cultures was observed in comparison to controls (Fig. 1B). To quantify the emergence of haematopoietic cells, the cultures were harvested at day 1, 2 and 3 and analysed for cell surface markers by flow cytometry. During embryonic haematopoiesis, haemangioblasts differentiate to haematopoietic stem and progenitor cells (HSPCs) through the intermediate haemogenic endothelium (HE)^8^,^9^,^13^. The HE cells express endothelial markers, such as TIE2 or VE-CADHERIN^40^. The subsequent generation of HSPCs is associated with the acquisition of CD41 expression, an early haematopoietic progenitor marker, by the HE followed by the loss of its endothelial markers. Our FACS analyses revealed that culture of haemangioblasts on GO, in comparison to GE, resulted in a higher frequency of CD41 positive cells at all the time points examined (Fig. 1C,D). In particular the frequency of HE cells double positive for endothelial (TIE2) and haematopoietic (CD41) markers was increased in the GO cultures. These results are consistent with the morphological changes observed through bright field microscopy. Importantly, we did not observe any significant difference in the total number of cells present at different time points in cultures on GE or GO (Fig. 1E). In addition, the positive effect mediated by GO on haematopoietic production was maintained with different initial seeding density of haemangioblast (data not shown). Taken together, these results suggest a positive role for GO in the generation of haematopoietic cells from mesodermal precursors.

### GO does not affect cell proliferation or survival of mesoderm-derived cells

**Figure 2:**
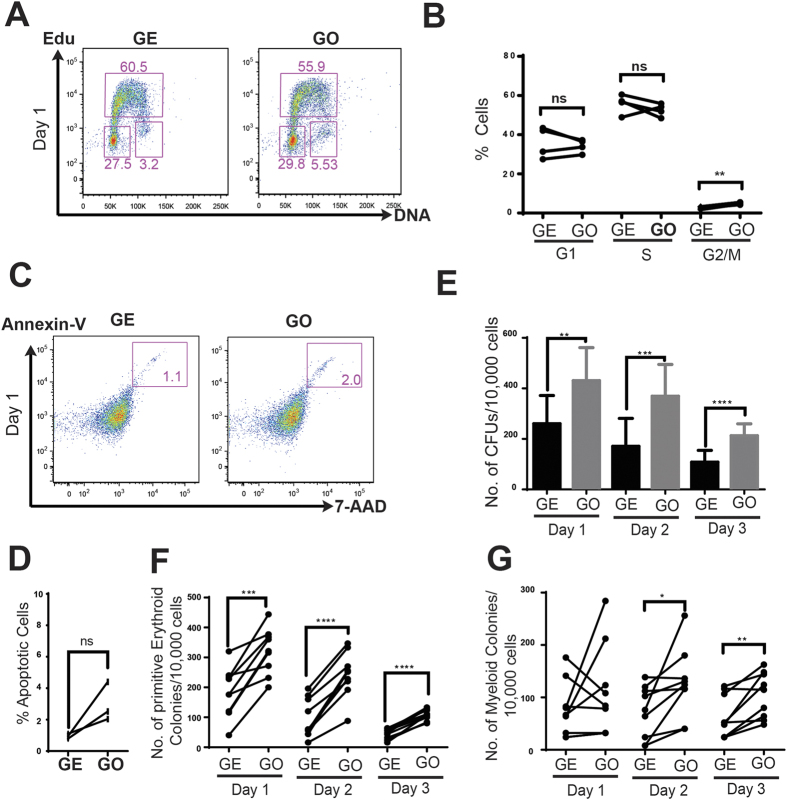
Effect of GO on cell cycle status and clonogenicity of haemangioblast cultures. (**A**) FACS detection of Edu incorporation. Day 1 haemangioblast cultures that were grown on GE or GO, were incubated with Edu for 4 hours prior to fixing. DNA content is shown on X-axis and Edu intensity is shown on Y-axis. (**B**) Percentage of haemangioblast cultures in G1, S and G2/M phases of cell cycle stage are shown (N = 4). (**C**) FACS analyses of day 1 haemangioblast cultures grown on either GE or GO for detection of apoptotic cells. (**D**) Percentage of apoptotic cells in haemangioblast cultures grown on either GE or GO are shown (N = 4). Total number of all haematopoietic (**E**), erythroid (**F**) and myeloid (**G**) colonies observed per 10,000 cells seeded from haemangioblasts cultures at indicated days grown either on GE or GO. Asterisks indicate significant differences (Paired t-test. **p < 0.01, ***p < 0.001, ****p < 0.0001) n.s. non significant.

We next wanted to investigate whether the increase in number of CD41^+^ cells generated in the presence of GO was either due to an enhanced production of these cells by the haemangioblasts or to altered cell cycle status and/or survival of these cells in these culture conditions. EdU incorporation assay was used to assess cell proliferation and cell cycle progression of the haemangioblast cultures. These analyses did not reveal any significant changes in EdU labelling indicating that haemangioblasts cultures on GO and GE proliferate at similar rate at day 1 (Fig. 2A,B). We did not detect any differences in the percentage of cells in G1 phase of cell cycle. Although we measured a small, but significant increase in number of cells in G2/M phase of the cell cycle in cultures on GO, we could not attribute any functional significance to this observation. Together these results indicate that GO coated-plates does not significantly affect cell proliferation during the generation of haematopoietic cells in haemangioblast cultures. In order to address whether GO promotes cell survival by inhibiting apoptosis, we quantified the number of apoptotic cells in day 1-haemangioblast cultures. Our results showed a marginal but not significant increase in the number of apoptotic cells as determined by annexin V staining and 7AAD incorporation when cultured on GO (Fig. 2C,D). Collectively our results suggest that GO does not enhance haematopoietic lineage specification through an increase in cell proliferation or survival.

### Functionality of GO induced mesoderm derived blood cells

Our data indicated that culture of haemangioblasts on GO, in comparison to GE, resulted in a higher production of CD41^+^ cells at all time points examined (Fig. 1C,D). To assess if these CD41^+^ cells are functional haematopoietic progenitors, the blast cultures were harvested at day 1, 2, and 3, dissociated and replated in clonogenic assays in semi-solid methylcellulose cultures containing haematopoietic cytokines to quantify colony-forming units (CFUs). Cultures previously maintained on GO generated a higher number of haematopoietic colonies at all time points examined (Fig. 2E). Embryonic haematopoiesis produced by haemangioblasts results in the generation of both primitive erythroid and definitive haematopoietic progenitors^8^,^9^,^41^. We observed a significant increase in the number of primitive erythroid colonies generated from cultures grown on GO (Fig. 2F). A more modest increase in number of myeloid colonies was also observed from haemangioblast cultures grown on GO (Fig. 2G).

**Figure 3:**
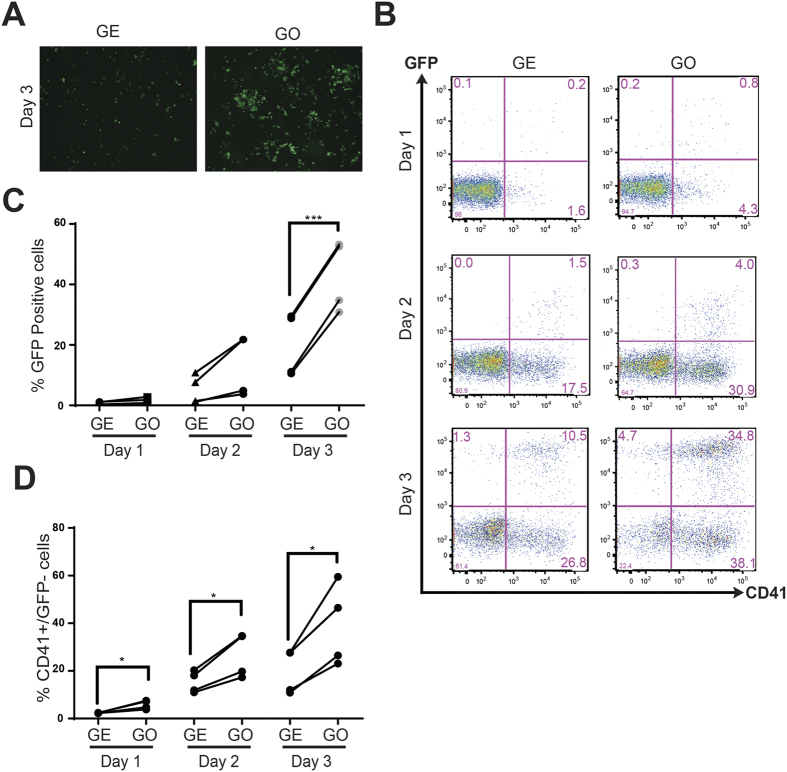
GO enhances both primitive and definitive haematopoiesis. (**A**) Fluorescent microscopic images of GFP positive cells in haemangioblasts cultures at day 3 grown on either GE or GO. (**B**) FACS analyses of haemangioblast cultures grown on either GE or GO at indicated days for GFP and CD41expression. (**C**) Percentages of GFP positive cells in haemangioblast cultures cultured on either GE or GO at indicated days (N = 4). (**D**) Percentages of GFP negative and CD41 positive cells in haemangioblast cultures cultured on either GE or GO at indicated days (N = 4). Scale bars represent 100 μm. Asterisks indicate significant differences (Paired t-test. *p < 0.05, ***p < 0.001).

To further examine the role of GO in the induction of haematopoiesis, we made use of a βH1-GFP reporter ESC line containing a bacterial artificial chromosome (BAC) in which the green fluorescent protein (GFP) encoding gene has been knocked-in the embryonic βH1 haemoglobin locus, which is specifically transcribed in primitive erythroid cells. As shown in Fig. 3A, higher number of GFP positive cells were detected by fluorescence microscopy in cultures on GO in comparison to GE. Accordingly, we detected higher frequencies of GFP^+^ cells at all time points analysed by flow cytometry (Fig. 3B). On average, we observed over a two-fold increase in the presence of primitive erythroid cells at day 3 of the cultures (Fig. 3C). Together these results clearly indicate that GO promotes primitive erythropoiesis and are consistent with the higher numbers of primitive erythroid colonies generated by GO cultures.

**Figure 4:**
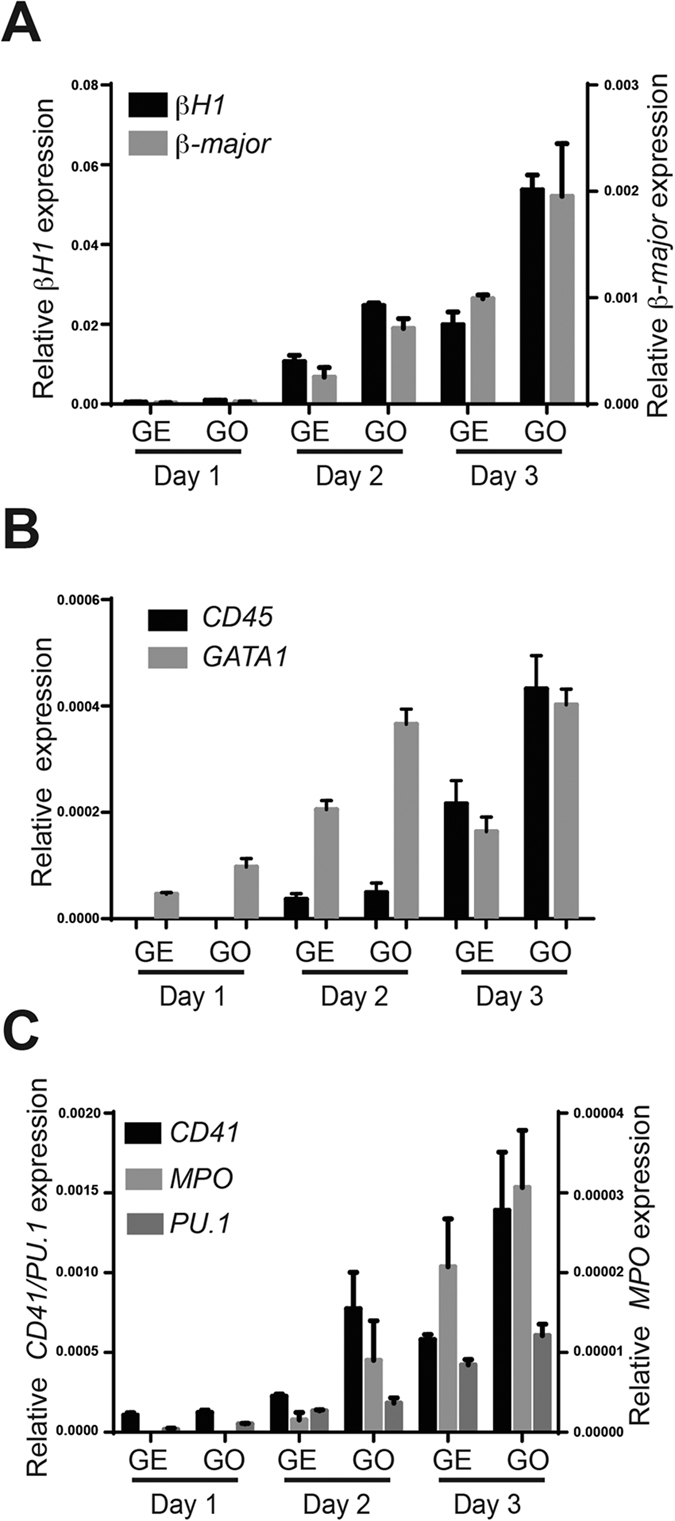
GO induces haematopoietic gene expression in haemangioblast cultures. (**A–C**) Relative gene expression levels of haematopoietic markers with respect to β-actin at indicated days of haemangioblast cultures grown on GE or GO. Data represented are mean ± SD from one representative experiment (N = 3).

To investigate whether GO also induces definitive haematopoiesis, we quantified the frequency of CD41^+^ cells that are negative for GFP and therefore correspond to definitive haematopoietic cells. Again the frequency of GFP^−^CD41^+^ cells was significantly higher when cultured on GO (Fig. 3D). Collectively these results indicate that GO promotes the induction of both primitive as well as definitive haematopoiesis. To further validate these data we performed QRT-PCR analyses for the expression of haematopoietic genes. These analyses revealed an increase in the expression of both primitive (βH1) as well as definitive (β-major) erythroid markers when cultured on GO (Fig. 4A). We also detected increased expression of the haematopoietic genes *Pu.1, MPO, CD45*, and *Gata1* confirming the role of GO in promoting haematopoiesis (Fig. 4B,C). Altogether these results establish that GO increases the generation of both primitive and definitive haematopoietic cells from haemangioblasts compared to GE.

### Haemangioblast to HE transition is induced by GO

**Figure 5:**
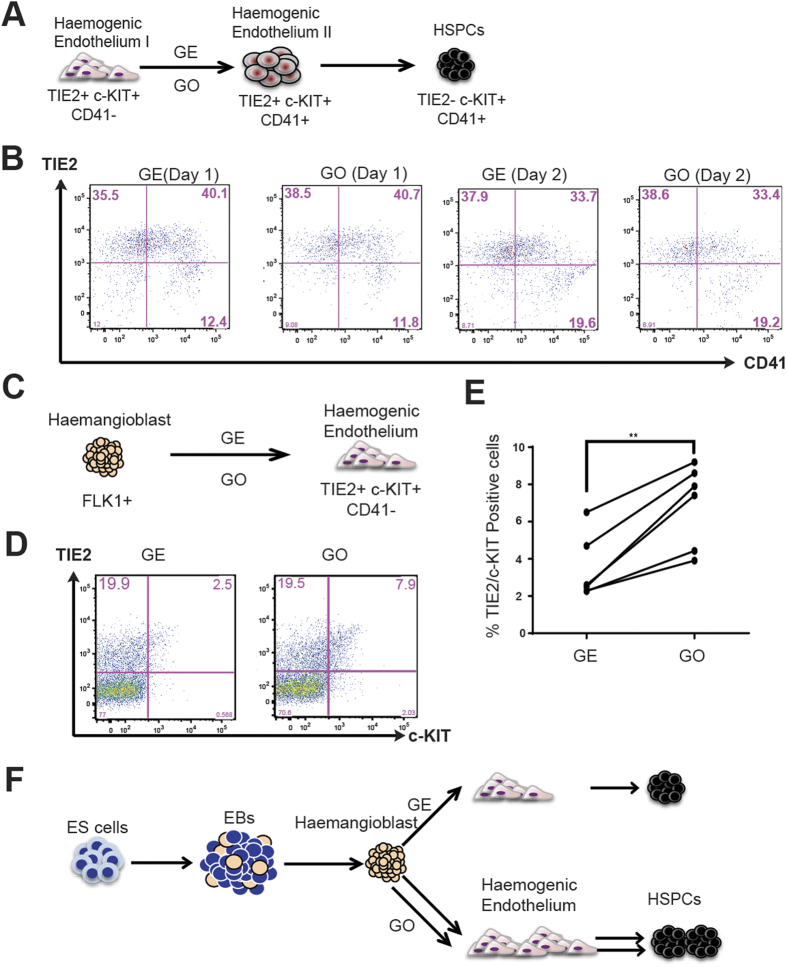
GO enhances Haemangioblasts to HE transition. (**A**) Schematic representation of experimental strategy. Haemogenic Endothelium I (HEI) cells were sorted from 2 day old haemangioblasts and were further cultured on either GE or GO. FACS analyses were performed to detect HE I, HE II and HSPC populations. (**B**) FACS analyses for indicated markers of HE cultures grown on either GE or GO at indicated days. (**C**) Schematic representation of experimental strategy. Haemangioblasts were cultured either on GE or GO for 1 day. FACS analyses were performed to detect HEI population. (**D**) FACS analyses of haemangioblasts grown on either GE or GO at day1. (**E**) Average percentages of TIE2/c-KIT double positive cells in haemangioblast cultures grown on either GE or GO at day 1 (N = 6). Asterisks indicate significant differences (Paired t-test. **p < 0.01). (**F**) Schematic representation of mechanism of action of GO. GO induces the transition of haemangioblast to haemogenic endothelium thereby promotes the generation of HSPCs.

Blood progenitors emerge from the HE through a process known as endothelial to haematopoietic transition (EHT)^42^. To gain further insights into the mechanisms by which GO promotes haematopoiesis, we investigated the role of GO on EHT. The earliest haemogenic endothelial cell population (HE I) expresses the endothelial marker TIE2 and progenitor marker c-KIT, but lacks the expression of the early haematopoietic marker CD41. During the EHT transition the cells become first positive for CD41 (HE II) before losing the expression of TIE2 (Fig. 5A)^43^. To evaluate the effect of GO on the EHT, the HE I (TIE2^+^ c-KIT^+^ and CD41^−^) cell population was sorted from 2-day haemangioblasts cultured on GE and seeded on either GE or GO-coated cover slips. The transition to the haematopoietic lineage was monitored by FACS for gain of CD41 expression and loss of TIE2 expression. As shown in Fig. 5B, we did not detect any significant changes in the kinetic or frequency of this transition when HE I was cultured on GO. Together these results indicate that GO does not affect the EHT process and suggest that GO is acting before the transition of HE I to haematopoietic cells i.e. during haemangioblast to HE I transition.

To evaluate this hypothesis, we examined the emergence of TIE2^+^/c-KIT^+^ (HE I) cells from FLK1^+^ haemangioblast cell population cultured on GO or GE (Fig. 5C). We observed a significant increase in the frequencies of emergence of these cells within the GO cultures, providing direct evidence for a positive effect of GO in the generation of haemogenic endothelial cells (Fig. 5D,E). Additionally, we did not detect any change in cell cycle kinetics of TIE2^+^/c-KIT^+^ cells when cultured on GO (data not shown). Taken together our results show that GO promotes the transition of haemangioblasts to haemogenic endothelium and thereby positively influence both primitive as well as definitive haematopoiesis (Fig. 5F).

### GO promotes transition of human ES cell derived haemangioblasts to haemogenic endothelium

**Figure 6:**
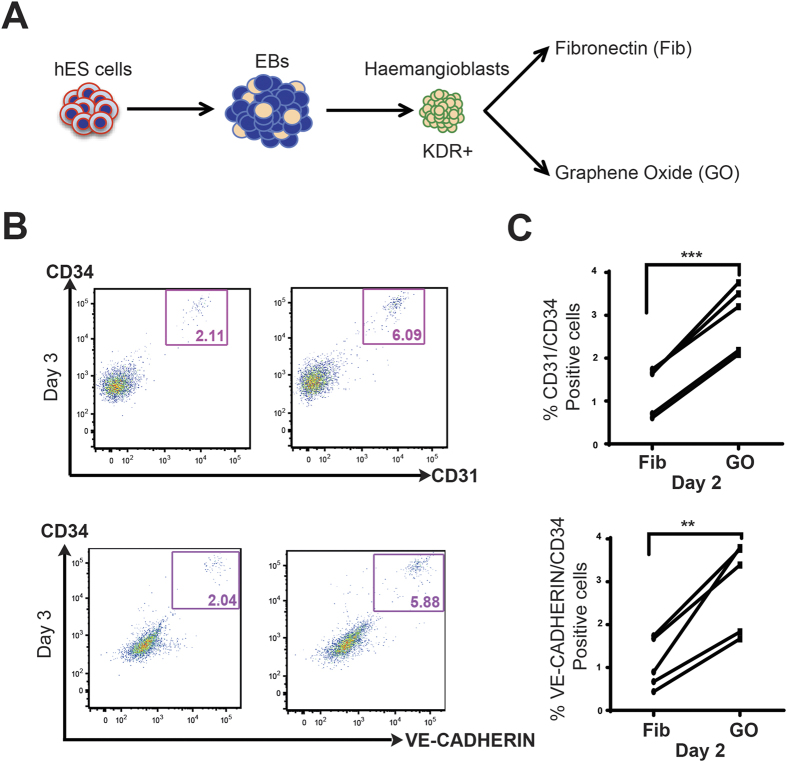
Role of GO on human haemogenic endothelial cell fate. (**A**) Schematic representation of experimental strategy. Haemangioblasts were cell sorted from 4-day-old human ES derived embryoid bodies and were seeded on to either fibronectin (Fib) coated plastic plates or graphene oxide (GO) coated cover slips. Cells were harvested at day 1, 2 and 3 and FACs analysed for markers CD31, VE-CADHERIN and CD34. (**B**) FACS analyses of human haemangioblast cultures at day 3 grown on either Fib or GO. (**C**) Percentages of CD31/CD34 or VE-CADHERIN/CD34 positive cells in haemangioblast cultures grown on either Fib or GO at day 2 (N = 3). Asterisks indicate significant differences (Paired t-test. ***p < 0.001, **p < 0.01).

We next wanted to investigate whether GO exerts a similar positive effect on haematopoietic cell generation from human ESC. Human ESCs were differentiated as embryoid bodies for 4 days and haemangioblast population was sorted based on the expression of kinase-insert domain-containing receptor (KDR), the human equivalent of murine FLK1. The human haemangioblasts were either cultured on GO or control fibronectin (Fib) (Fig. 6A). In humans, the expression of CD31, VE-CADHERIN and CD34 defines haemogenic endothelial cells^44^. We observed higher frequencies of CD31/CD34 and CD34/VE-CADHERIN double positive haemogenic endothelial cells in cultures on GO at day 3 and day 2 (Fig. 6B,C). Altogether, these results indicate that GO promotes the differentiation of both murine as well as human ESCs to haemogenic endothelial cells.

## Discussion

In this manuscript, we evaluated the potential influence of GO-coated cell culture substrate on the differentiation of murine ESCs to haematopoietic progenitor cells. Our results indicate that cultures on GO-coated plates result in a significant increase in the production of primitive and definitive haematopoietic cells upon i*n vitro* differentiation. We further demonstrate that this increase in blood cells production stems from a higher rate of transition from murine haemangioblasts to haemogenic endothelium. Significantly, we showed that GO similarly promotes the transition of hESC derived haemangioblasts to haemogenic endothelial cells.

GO presents several physiochemical properties such as ultra-large surface areas, abundant hydrophilic groups allowing bio-functionalization, and outstanding water solubility that makes it a promising biomaterial in a plethora of application in biotechnology such as drug carriers, biosensors, and antibacterial agents. Our study complements previous works that indicated a beneficial effect of using GO-based biomaterials in the differentiation of pluripotent, mesenchymal and neuronal stem cells^33^,^35^,^36^,^37^. The underlying biochemical processes behind the benefits of using GO over GE for the promotion of haematopoietic programme remains unclear but it is likely to arise from GO’s unique physicochemical properties. GO has been shown to modulate WNT, Notch, STAT1/3 and NRF2 pathways in MCF7 cells^38^. Temporal action of WNT and Notch pathways selectively regulate haematopoietic specification^45^. It would be therefore interesting to investigate to what extent these WNT/Notch signalling pathways are differentially modulated in haemangioblast cultures grown on GO. GO mechanically modulate cell adhesion, cell-cell interaction, and cell spreading owing to its different surface characteristics such as mechanical stiffness, nanotopography and large absorption capacity^46^. Additional studies at molecular level need to be performed to further elucidate whether GO induces changes in cellular morphology of haemangioblast cultures and whether such changes in cell morphology contribute to induction of haematopoiesis. GO is also internalized via clathrin-mediated endocytosis and intracellularly triggers specific signalling pathways. However this mode of action does not apply to our study as graphene oxide is on a coated surface. Reduced-Graphene Oxide/Gelatin (rGO/GE) composites have been shown to have increased tensile strength, better-wet stability and minimal cytotoxicity^47^. Therefore, it would be interesting to evaluate similarly these rGO/GE composite films in ESC differentiation to blood cells.

Increasing evidence indicates that there is a critical need for micro environmental optimization for efficient maintenance and differentiation of both adult and embryonic stem cells^48^,^49^. In this regard, biomaterials have received much attention for their ability to create tissue mimetic microenvironment^50^. Modulating microenvironment by means of biomaterials in addition to growth factors and cytokines could have pivotal significance in tissue engineering for regenerative medicinal purposes^51^. In addition, i*n vitro* recapitulation of complexes environments such as the haematopoietic stem cells (HSC) niche will probably require the identification and combination of several biomaterials that mimic the complex microenvironment in which HSCs reside. Along this line, Liesten *et al*. have shown that a 3D environment containing collagen and bone marrow derived MSCs can promote expansion of haematopoietic progenitor cells^52^. Recently, a 3D bone marrow model consisting of porous silk has been shown to be functional in generating platelets *ex vivo* upon co-culture with endothelial cells^53^. Furthermore, graphene based bio-scaffold allow the efficient differentiation of MSCs to bone cells pointing to their potential use in bone marrow mimetics^54^. Finally, studies indicate that GO could maintain cord blood haematopoietic progenitors with minimal toxicity indicate a good biocompatibility of GO sheets^32^. Accordingly, we did not observe any GO induced cytotoxicity on haemangioblast cultures derived from murine ESCs (Fig. 2). Therefore, culturing endothelial cells or MSCs along with GO based materials could mimic biocompatible bone marrow niche and be helpful in generating the relevant number of haematopoietic cells required for clinical regenerative medicine purposes. Additionally 3D scaffolds composed of GO and polypeptide thermogels/porcine acellular dermal matrix or 3D graphene foam could provide the necessary bioactive cues to maintain and expand haematopoietic cells^55^.

Our study demonstrates a beneficial effect of GO cell culture substratum on haematopoietic lineage specification in ESC differentiation system. Our results indicate that this positive effect does not result from an increase in EHT or cell proliferation, but is due to the ability of GO to promote haemangioblast to HE transition. Furthermore our results also suggest that substrate dependency varies between different stages of stem cell differentiation to blood. Importantly, similar effects were observed with hESC derived haemangioblasts (Fig. 6). The promotion of both primitive as well as definitive haematopoiesis by GO is consistent with the fact that primitive as well as definitive progenitors are generated from HE precursors. As definitive haematopoiesis generates the most clinically relevant adult cell populations (HSCs, red blood cells, platelets, B and T cells), future efforts should be aimed at promoting this developmental pathway. Functionalizing GO with other bio-active molecules is a promising strategy that would not only help to direct efficient differentiation but also start to create 3D scaffolds modelling bone marrow niche. Composites of graphene and carbon nanotubes have been shown to be advantageous for biomedicinal applications such as cell adhesion, proliferation, cancer cell damage and DNA binding^56^. Collectively these results highlight the importance of graphene-based biomaterials in the future expansion protocols for cellular therapies and transplantations.

## Materials and Methods

### Preparation and characterization of GO

Graphite oxide was prepared according to a modified Hummers method^57^ and exfoliated to yield GO. To prepare glass substrates, coverslips were cleaned by sonication for 15 minutes in acetone, DI water and isopropanol, followed by nitrogen drying and 10 min treatment in oxygen plasma to render the surface hydrophilic. The GO deposition was done by spin coating: 200 μl of GO dispersion of 2.3 mg/ml were spin coated on freshly cleaned and treated substrates, using a speed of 3000 rpm, 300 rpm/sec acceleration and 150 sec spinning time. The GO covered substrates were baked at 120 °C for 5 min in air to ensure the stability of the film. Samples for SEM and AFM analysis of the GO flakes were prepared by spin coating on to silicon wafer pieces. Samples for XPS and Raman spectroscopy were prepared by drop-casting a thick film of GO on silicon wafer pieces and drying.

### Cell Culture and EB differentiation

Murine embryonic stem cells were differentiated to haemangioblasts as described previously^43^. Haemangioblasts were FACS sorted based on FLK1 expression. Haemangioblasts were cultured in blast medium containing 1× IMDM, 10% FBS, 0.5 mM Ascorbic Acid, 4.5 × 10 − 4 M MTG, 2 mM L-glutamine, 80 mg/ml transferrin and 50 μg/ml penicillin-streptomycin. Haemangioblast cultures were harvested at indicated days for phenotypic analyses. Haemogenic endothelial cells were sorted based on the expression of TIE2, c-KIT and CD41 from 2-day old haemangioblast cultures. Haemogenic endothelial cells were cultured in haematopoietic media containing 1X IMDM supplemented with plasma-derived serum (PDS;Antech), 10% protein-free hybridoma medium (PFM; Gibco), 0.5 mM Ascorbic Acid, 4.5 × 10 − 4 M MTG, 2 mM L-glutamine, 80 mg/ml transferrin, 50 μg/ml penicillin-streptomycin in addition to the following specific cytokines/growth factors; 1% IL3, 1% of GM-CSF, 10 ng/ml M-CSF 1% c-KIT ligand, 1% Thrombopoietin, 10 ng/ml 5 ng/ml IL11, IL6 and 4 U/ml Erythropoietin (Ortho-Biotech). Haemogenic endothelial cell cultures were harvested at day 1 and 2 and analysed by FACS for the indicated cell surface markers.

Man-5 is a research grade hESC line derived by the UK North West Embryonic Stem Cell Centre^58^. Man-5 cells were maintained on γ-irradiated MEF feeders in hESC basal medium as described previously^59^. For passaging, nearly confluent hESCs were dissociated by non-enzymatic methods using 50 mM EDTA/PBS solution as previously described^60^. Prior to EB differentiation, hESCs were plated for one passage on Geltrex LDEV-Free Reduced Growth Factor Basement Membrane Matrix (Life Technologies)-coated plates in TeSR™-E8™ defined medium (Stem Cell Technologies) to remove MEF feeders.

### EB differentiation of hESC

Nearly confluent hESCs, maintained in feeder free conditions, were treated with 50 mM EDTA/PBS solution for 1 min at 37 °C and upon addition of defined medium, the cells were manually cut with StemPro EZ Passaging Tool (Life Technologies) to obtain similar size pieces of colonies to generate homogenous sizes of self-aggregated EBs. Cell clumps collected with serological pipette were left to settle down at room temperature and supernatant was removed. Aggregates were resuspended in serum free StemPro^®^-34 SFM media (Thermo Fisher) supplemented with 0.5% penicillin/streptomycin, L-glutamine (2 mM), ascorbic acid (1 mM), monothioglycerol (4 × 10 − 4 M; Sigma-Aldrich), transferrin (150 mg/ml) and BMP-4 (10 ng/ml), seeded into low attachment dishes (Corning) to generate embryoid bodies under hypoxic conditions for 4 days. Human haemangioblasts were FACS sorted based on KDR expression and further cultured on fibronectin or GO coated cover slips in serum free StemPro^®^-34 SFM media (Thermo Fisher) supplemented with 0.5% penicillin/streptomycin, L-glutamine (2 mM), ascorbic acid (1 mM), monothioglycerol (4 × 10 − 4 M; Sigma-Aldrich), 150 mg/ml transferrin, 10 ng/ml VEGF, 5ng/ml bFGF, 50 ng/ml hSCF, and hIL6 (10 ng/ml) (PropsecBio).

### Flow cytometry

Cells harvested at indicated times were stained with following antibodies: mFLK1-APC (1560), hKDR-Alexafluor 647 (Biolegend-338909), hCD31-APC Cy7 (WM-59), hCD34-FITC (AC 136), CD41-PE-Cy7 (MWReg30), mc-KIT-APCeFluor780 (2B8), mTIE2-PE (eBio12-5987), mCDH5–APC (eBioBV13), mCD45-PerCp5.5 (30-F11;Biolegend). Stainings were done in 50-ul-reaction volume for 30 minutes on ice. Compensations were performed with beads controls. Acquisition was done on LSRII or Fortessa (BD Biosciences) and data was analysed using FlowJo software (Treestar). Cell sortings were performed on AriaII or AriaIII or Influx sorters (BD Biosciences).

### Haematopoietic CFU-C assay

Haematopoietic colony forming potentials of cells were assessed in semi solid methylcellulose cultures. 10,000 cells at indicated time points were plated into haematopoietic media containing 1% methylcellulose. The number of primitive erythroid colonies was scored after 4 days of incubation at 37 °C and 5% CO2. Granulocytic and macrophagic colonies were scored after 7 days of incubation.

### Quantitative RT-PCR analysis

Total RNAs were isolated using QIAGEN RNA Prep KIT (Qiagen). cDNAs were generated using Omni Script (Qiagen) according to manufacturer’s protocols. Taqman probes were used to detect relative levels of indicated genes.

### Cell cycle and apoptosis analysis

Cell cycle analyses were performed using the Click-iT Edu flowcytometry assay kit as per manufacturer’s guidelines (Invitrogen). Apoptosis assays were performed using PE-AnnexinV apoptosis detection kit as per manufacturer’s guidelines (BD Pharmigen).

## Additional Information

**How to cite this article**: Garcia-Alegria, E. *et al*. Graphene Oxide promotes embryonic stem cell differentiation to haematopoietic lineage. *Sci. Rep.* **6**, 25917; doi: 10.1038/srep25917 (2016).

## Supplementary Material

Supplementary Information
