# Corrigendum: Graphene Oxide promotes embryonic stem cell differentiation to haematopoietic lineage

**DOI:** 10.1038/srep28723

**Published:** 2016-07-04

**Authors:** Eva Garcia-Alegria, Maria Iliut, Monika Stefanska, Claudio Silva, Sebastian Heeg, Susan J. Kimber, Valerie Kouskoff, Georges Lacaud, Aravind Vijayaraghavan, Kiran Batta

Scientific Reports 6: Article number: 2591710.1038/srep25917; published online: 05202016; updated: 07042016

The original version of this Article contained a typographical error in the spelling of the author Maria Iliut which was incorrectly given as Maria Iluit. This has now been corrected in the PDF and HTML versions of the Article.

