# Supplementary material for: Graphene Oxide promotes embryonic stem cell differentiation to haematopoietic lineage
Source: Sci Rep. 2016 May 20;6:25917. doi: 10.1038/srep25917 (PMC4873758; doi:10.1038/srep25917)
Supplement: Supplementary Information [file srep25917-s1.pdf]

Graphene Oxide promotes embryonic stem cell differentiation to haematopoietic lineage

Eva Garcia-Alegria[1], Maria Iluit[2], Monika Stefanska[3], Claudio Silva[2], Sebastian Heeg[2], Susan J. Kimber[4], Valerie Kouskoff[1]* Georges Lacaud[3]*, Aravind Vijayaraghavan[2]*, and Kiran Batta[3]*

Supplementary material and methods:

Figure S1: (A) Photograph of glass cover slip uncoated (left) and coated (right) with GO **(B)** Dark field optical micrograph and **(C)** atomic force micrograph of GO coating on cover slip demonstrating uniformity of coverage. **(D)** AFM image of a scratch marked across the GO coating to measure coating height (inset).

Figure S2: (A) SEM image of GO flakes spin-coated on a SiO_2 substrate to measure lateral dimension distribution, and a bottle of GO dispersion (inset). **(B)** AFM image of individual GO flake showing monolayer thickness.

Figure S3: (A) XPS spectrum of the C1s core level showing components, **(B)** Raman spectrum showing G- and D- peaks and **(C)** UV-Vis-NIR absorption spectrum showing the π and π* transitions of GO.

Figure S4: (A) 8 additional AFM images and (B) 5 additional SEM images of GO flakes spin-coated on to SiO_2 substrate.

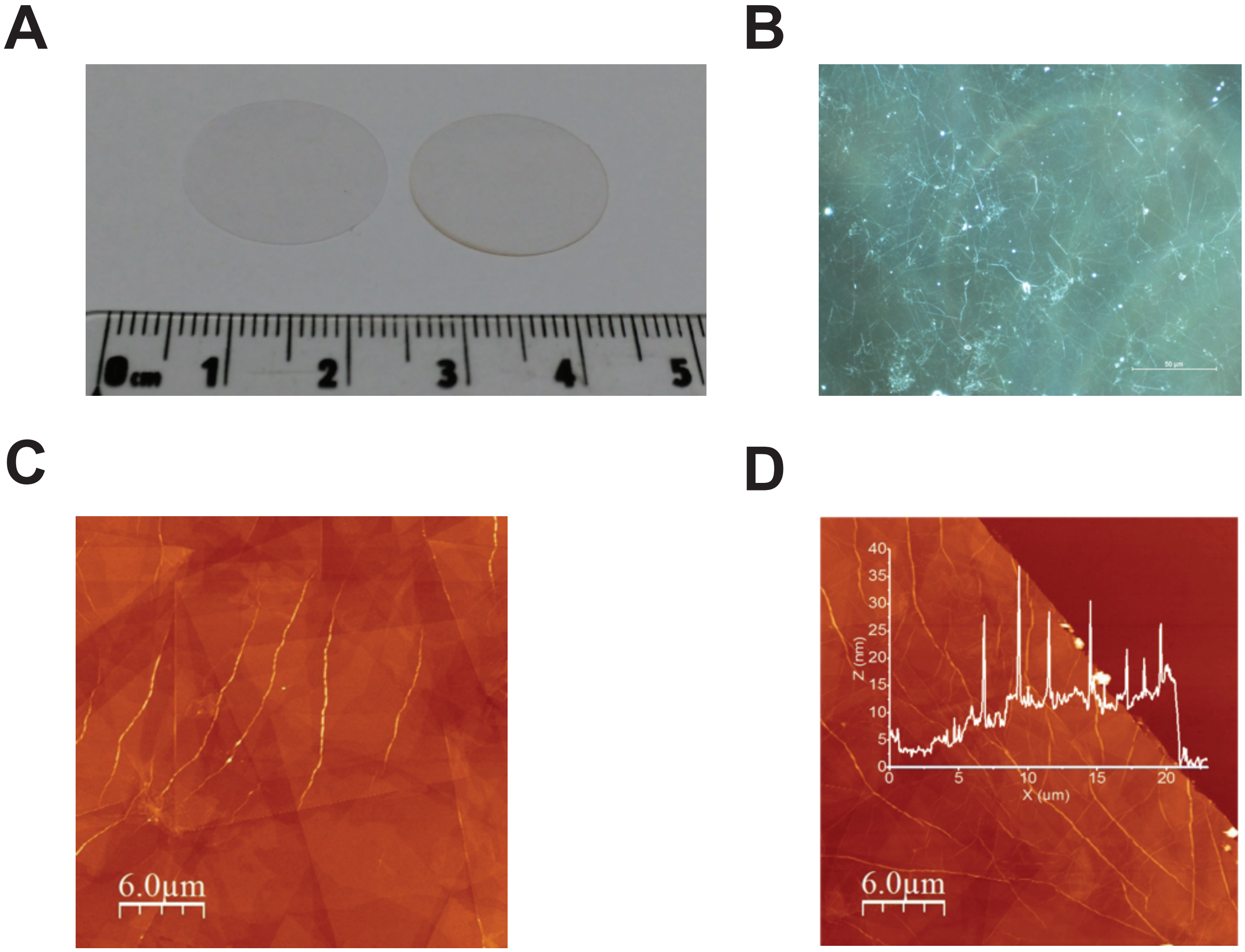
A
B
C
D
0cm 1 2 3 4 5
6.0μm
6.0μm
Z (nm)
0 5 10 15 20 25 30 35 40
X (um)
0 5 10 15 20

S. Figure 1

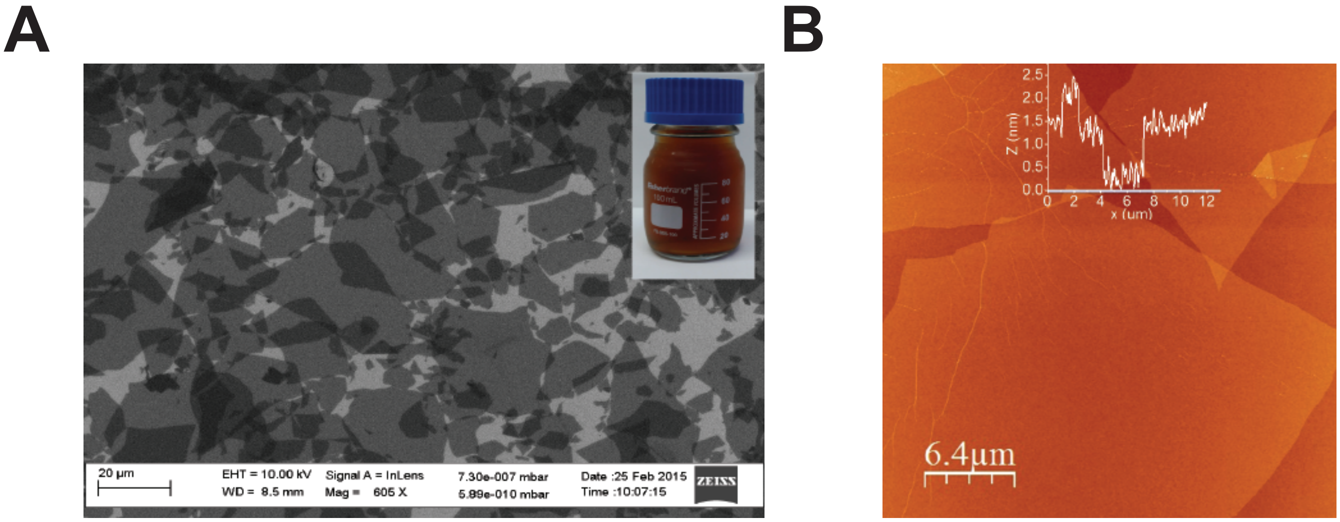
A
B
20 µm
EHT = 10.00 kV
WD = 8.5 mm
Signal A = InLens
Mag = 605 X
7.30e-007 mbar
5.89e-010 mbar
Date :25 Feb 2015
Time :10:07:15
ZEISS
Z (nm)
2.5
2.0
1.5
1.0
0.5
0.0
0 2 4 6 8 10 12
x (um)
6.4µm

S. Figure 2

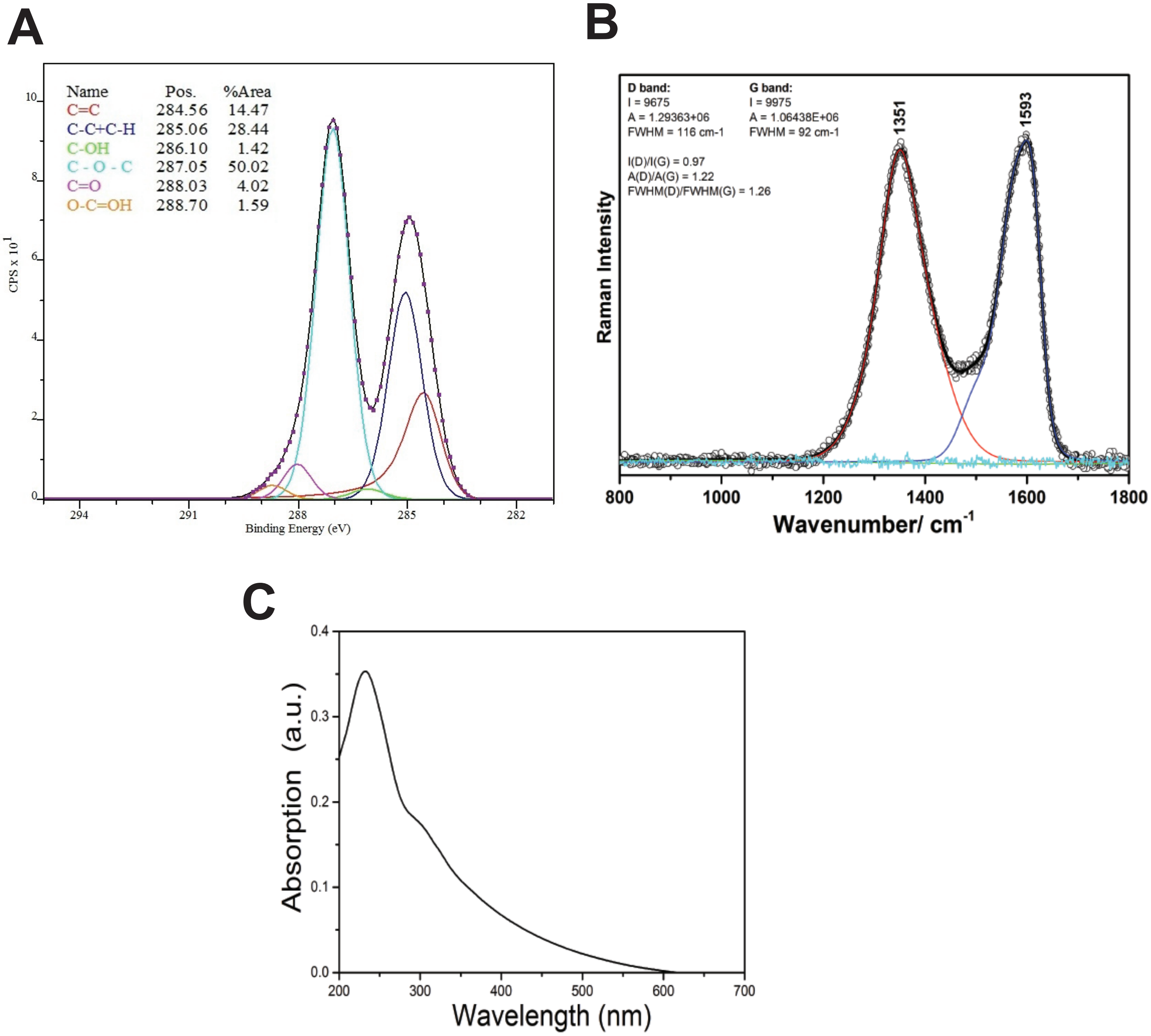
A
Name Pos. %Area
C=C 284.56 14.47
C-C+C-H 285.06 28.44
C-OH 286.10 1.42
C - O - C 287.05 50.02
C=O 288.03 4.02
O-C=OH 288.70 1.59
CPS x 10^1
Binding Energy (eV)
294 291 288 285 282
0 2 4 6 8 10
B
D band:
I = 9675
A = 1.29363+06
FWHM = 116 cm-1
G band:
I = 9975
A = 1.06438E+06
FWHM = 92 cm-1
I(D)/I(G) = 0.97
A(D)/A(G) = 1.22
FWHM(D)/FWHM(G) = 1.26
1351
1593
Raman Intensity
Wavenumber/ cm-1
800 1000 1200 1400 1600 1800
C
Absorption (a.u.)
Wavelength (nm)
0.0 0.1 0.2 0.3 0.4
200 300 400 500 600 700

S. Figure 3

A

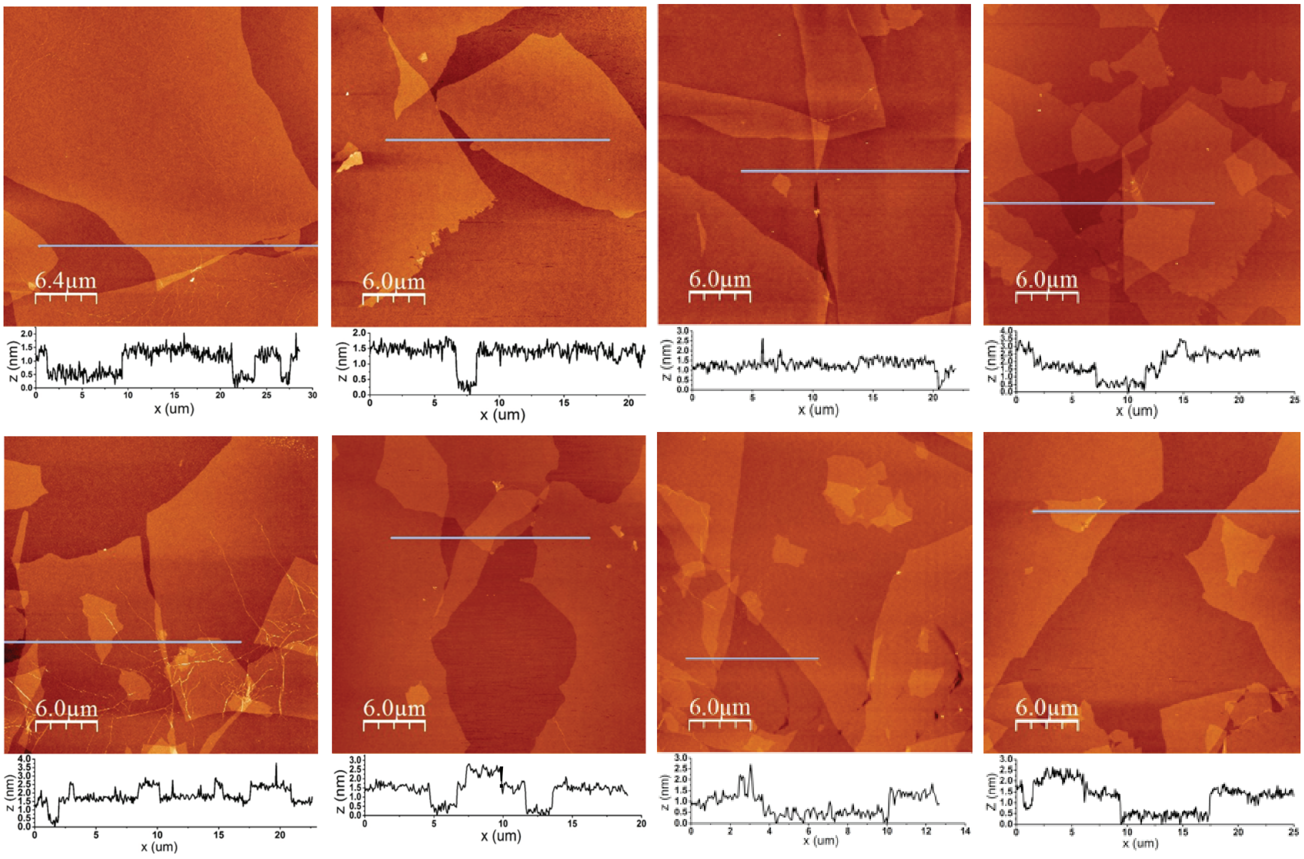
6.4μm
6.0μm
z (nm)
x (um)

B

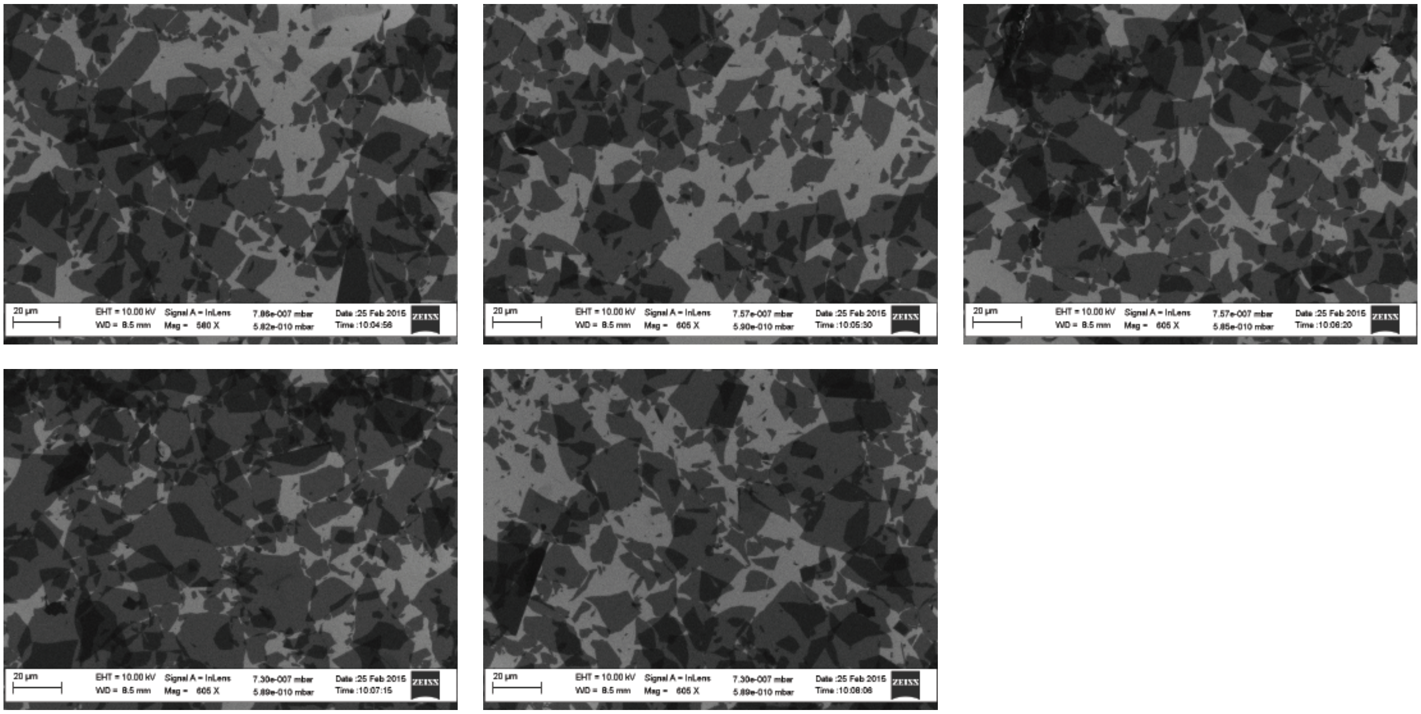
20 μm
EHT = 10.00 kV
Signal A = InLens
Date :25 Feb 2015

S. Figure 4
